# Temporal Dynamics of Interferon Gamma Responses in Children Evaluated for Tuberculosis

**DOI:** 10.1371/journal.pone.0004130

**Published:** 2009-01-06

**Authors:** Jean-Louis Herrmann, Marie Belloy, Raphael Porcher, Nancy Simonney, Rola Aboutaam, Muriel Lebourgeois, Joel Gaudelus, Laure De LosAngeles, Katarina Chadelat, Pierre Scheinmann, Nicole Beydon, Brigitte Fauroux, Martine Bingen, Mustapha Terki, Dominique Barraud, Philippe Cruaud, Catherine Offredo, Agnes Ferroni, Patrick Berche, Didier Moissenet, Hoang Vuthien, Catherine Doit, Edouard Bingen, Philippe Henri Lagrange

**Affiliations:** 1 Service de Microbiologie, Hôpital St Louis et Equipe d'Accueil (EA3510), Assistance Publique Hôpitaux de Paris et UFR Denis Diderot, Paris, France; 2 Service de Pneumologie Pédiatrique et Fédération de Biologie, Centre Hospitalier de Gonesse, Gonesse, France; 3 Département de Biostatistiques et Informatique Médicale, Hôpital Saint Louis, Assistance Publique Hôpitaux de Paris et UFR Denis Diderot, Paris, France; 4 Service de Pneumologie Pédiatrique et Service de Microbiologie, Hôpital Necker-Enfants Malades, Assistance Publique Hôpitaux de Paris et UFR Paris V, Paris, France; 5 Service de Pédiatrie Générale et Service de Microbiologie, Hôpital Jean Verdier, Bondy, France; 6 Service de Pédiatrie et Service de Microbiologie, Hôpital R Debré, Assistance Publique Hôpitaux de Paris et UFR Denis Diderot, Paris, France; 7 Service de Pneumologie Pédiatrique et Service de Microbiologie, Hôpital A Trousseau, Assistance Publique Hôpitaux de Paris et UFR Paris VI, Paris, France; University of Stellenbosch, South Africa

## Abstract

**Background:**

Development of T-cells based-Interferon gamma (IFNγ) assays has offered new possibilities for the diagnosis of latent tuberculosis infection (LTBI) and active disease in adults. Few studies have been performed in children, none in France. With reference to the published data on childhood TB epidemiology in the Paris and Ile de France Region, we considered it important to evaluate the performance of IGRA (QuantiFERON TB Gold *In Tube*®, QF-TB-IT) in the diagnosis and the follow-up through treatment of LTBI and active TB in a cohort of French children.

**Methodology/Principal Findings:**

131 children were recruited during a prospective and multicentre study (October 2005 and May 2007; Ethical Committee St Louis Hospital, Paris, study number 2005/32). Children were sampled at day 0, 10, 30, 60 (except Healthy Contacts, HC) and 90 for LTBI and HC, and a further day 120, and day 180 for active TB children. Median age was 7.4 years, with 91% of the children BCG vaccinated. LTBI and active TB children undergoing therapy produced significant higher IFNγ values after 10 days of treatment (p = 0.035). In addition, IFNγ values were significantly lower at the end of treatment compared to IFNγ values at day 0, although the number of positive patients was not significantly different between day 0 and end of treatment.

**Conclusions/ Significance:**

By following quantitative IFNγ values in each enrolled child with LTBI or active TB and receiving treatment, we were able to detect an increase in the IFNγ response at day 10 of treatment which might allow the confirmation of a diagnosis. In addition, a decline in IFNγ values during treatment makes it possible for clinicians to monitor the effect of preventive or curative therapy.

## Introduction

*Mycobacterium tuberculosis* (MTB) still represents one of the most important killers among infectious pathogens. 9.2 million new tuberculosis (TB) cases in the world were reported in 2006 giving a total prevalence of 14.2 million of cases when previously diagnosed and cases undergoing treatment are included [1]. Many developing countries still notify the bulk of TB cases, which confer a major risk for susceptible individuals to become infected by tubercle bacilli. Children belong to this category of susceptible individuals. Younger children are the most likely to develop active TB after contact with an active TB case, and by so doing are considered key indicators of MTB transmission in the community [2], [3]. The risk of developing active TB following acute infection is high (20–40%) in children aged between 0 and 5 years, compared to adults. The risk then decreases as age increases [2], [3].

However, childhood TB remains a neglected aspect of the TB epidemic because it is usually a paucibacillary acid fast smear negative disease, and is thus considered to make a relatively minor contribution to the spread of TB [4]–[6]. Childhood TB presents with a diversity of manifestations, both pulmonary and extrapulmonary. In addition, there is little prospect of achieving a widely available gold standard diagnosis for TB in children, either by means of microscopy, culture, PCR or serology [2], [7], [8]. Less than one half of active TB in children is culture proven, and PCR, despite its rapidity, is unable to improve on this [2]. At present, clinicians must rely on clinical criteria, the patient history, chest radiography and tuberculin skin testing (TST). None of these approaches is totally accurate, often leading to over diagnosis of TB in children who might then be unnecessarily treated for TB.

France belongs to the WHO Group 3 with an incidence rate 8.5/100,000 in 2006 [9]. However, this overall incidence rate may not be truly representative. Young adults and children less than 20 years old represent 10% of the TB case-load [9]. Paris and Ile de France Region, where our study was conducted possess an incidence rate nearly 3 times higher than our national incidence rate [9], and in several areas, close to incidence rate observed in some high burden countries described above. Without the assurance of an accurate diagnosis, the precise burden of childhood TB and its importance will remain uncertain and controversial.

The recent description and availability of Interferon-gamma release assays (IGRA); the QuantiFERON®-TB Gold *In-Tube* (QF-TB-IT, Cellestis, Australia) and the T-SPOT *TB* (T-SPOT®, Oxford Immunotec Ltd. UK); offers new possibilities for the diagnosis of latent TB infection (LTBI) and active TB in adults [10]. IGRA allow detection of circulating TB specific T-cells *in vitro* using either an “In Tube” whole blood or ELISPOT approach [10 and references therein]. By comparison, few studies have been performed in children [11]–[24]. In France only one adult study has been published so far [25] and none on childhood TB. Taking advantage of the ease of use with QF-TB-IT usage, and with reference to the published data on childhood TB epidemiology in the Paris and Ile de France Region, we considered it important to evaluate the performance of IGRA in the diagnosis of LTBI and active TB in a French children cohort in one of the highest TB incidence area in France. In addition, we evaluate IGRA by measuring the IFNγ response at day 10, 30, 60, 90 in treated LTBI children and an additional day 120 and 180 in treated active TB children. Results presented in this study allowed us to provide a comprehensive follow-up data on IFNγ response during treatment for latent TB infection and active TB disease in children.

## Methods

### Objectives

We conducted a prospective and multicentre study between October 2005 and May 2007, involving 5 hospitals located in Paris and North of Ile de France Region to evaluate the intrinsic performance of IGRA in a French children cohort with latent TB infection or active TB disease, and to monitor the IFNγ responses in these children, receiving antituberculous prophylaxis or therapy.

### Participants

Enrolled children were investigated according to the national guidelines published by La Société de Pneumologie de Langue Française [26] and Le Conseil Supérieur d'Hygiène Publique de France [27]. The study was performed blind as clinicians responsible were not provided the QF-TB-IT results throughout the study. The distribution of the children in the different groups, as described below, was performed by clinicians mainly based on patient history, clinical data, chest radiography, microbiological results and TST.

Healthy contacts were defined by clinicians as asymptomatic children with a suspicion of TB contact but with a negative TST or without a TST conversion, and normal chest radiography.

LTBI children were defined as any children for whom contact with an adult with TB is suspected, in addition to the different inclusion criteria described above and a positive TST (see below)

Active TB children were defined as any children with the above criteria and who had clinical signs and/or compatible chest radiographic abnormalities, and who responded to specific treatment for TB.

Exclusion criteria were active TB diagnosed and treated in the two years preceding the study, contact with a known TB adult case already treated for 3 months, recent travel in a highly endemic country, immunosuppression (HIV, transplant, treatment…) or any immune deficiency suspected or diagnosed and involving the humoral and cellular immunity, and finally absence of consent.

Control children were included based on strict inclusion criteria: children hospitalized for any disease other than tuberculosis, absence of known contact with an adult TB case, and signed consent by both parents and children. TST was not performed on control children (Ethical Committee recommendations), and only previous TST values (when known by the parents) were recorded. Control children were matched with age with latently infected or active TB children.

Children diagnosed with LTBI received chemoprophylaxis for a period of 3 months (Isoniazid+Rifampicin) and were followed up at day 0 (day of inclusion), 10, 30, 60 and 90 (end of treatment). Any child diagnosed with active TB was hospitalized and received chemotherapy (Isoniazid+Rifampicin+Pyrazinamide+/−Ethambutol) for the first two months and dual therapy (Isoniazid+Rifampicin) for the following four months. Children with active TB were followed up at day 0 (day of inclusion), 10, 30, 60, 90, 120, 180 (end of treatment). Finally, children considered as healthy contacts without LTBI were also followed up as for children with LTBI (except day 60) as nearly all received chemoprophylaxis (83%).

### Description of Procedures undertaken

The TST was performed by experienced nurses or paediatricians in antiTB centres called “Centre de Lutte Antituberculeuse” or in a medical ward organised in schools, or social centres, according to standard national guidelines using intradermal injection of 5 U of PPD (Tubertest ® Aventis Pasteur MSD, Lyon, France). The transverse induration was read at 72 h. TST was considered positive when: (i) a TST induration diameter ≥10 mm if non BCG vaccinated or BCG vaccinated but with a known close TB contact case; (ii) a TST induration greater than 15 mm if BCG vaccinated with unknown TB contact case; (iii) and TST conversion defined as an increase of TST induration diameter ≥10 mm when compared to a previous TST induration diameter measured before (mainly after BCG vaccination, when children entered nursery or are sent to school) [27]. Children suspected of being a TB contact with or without a positive TST, or who had clinical signs were then referred to the Pediatric hospital or a hospital with paediatric ward present in the same area or suburb, where clinicians participating in the study attended. TST was never done again at the hospital when the induration diameter was recently known.

The QF-TB-IT was sampled at day of inclusion (day 0) for all children. QF-TB-IT was then sampled by nurses or paediatricians during the normal follow-up and outpatient visit for the HC, LTBI (day 10, 30, 60 (except HC) and 90) and an additional day 120 and 180 for active TB children.

The supplier's recommendations were strictly followed. A positive test result was defined as >0.35 IU/ml. The QFT-TB-IT ELISA does not accurately determine IFN-γ concentrations greater than 10 IU/mL. Thus, to enable estimation of IFN-γ concentrations in plasma samples with values above 10 IU/ml, these samples were diluted 1/2 and 1/10 in the ELISA buffer reagent and retested in the ELISA. Concentrations were then calculated from the OD of the diluted plasma sample which fell within the dynamic range of the ELISA.

All children except controls, 2 HC, 17 LTBI and one active TB children had pulmonary (sputum, gastric aspirate, bronchoalveolar lavage…) and/or extra-pulmonary specimens when appropriate sent to the respective mycobacteriology laboratory.

### Ethics

The study was approved by the Saint Louis Hospital Ethical Committee (CCPPRB St Louis Hospital, Paris, study number 2005/32). Parents and children signed informed consent when accepting an invitation to participate in the study.

### Statistical analysis

Patient characteristics were reported as counts (percent) or median (range). Distributions of QF-TB-IT for all groups were represented using box and whiskers plots. As the distribution of QF-TB-IT was skewed, data were displayed on a log scale, and null values were arbitrarily set at 0.01 (below the smallest observed non-null value). QF-TB-IT positivity was defined as values equal or superior to 0.35 (which correspond to −0.46 on the log scale). These distributions were compared using Kruskal-Wallis tests and, in case of a significant difference, post-hoc two by two group comparisons were performed using Wilcoxon rank sum tests and Hochberg's method to correct p-values for multiple testing. Similar analyses were conducted after adjustment for age, using analysis of covariance of log-transformed data. The properties of QF-TB-IT as a diagnostic marker of TB infection were assessed using the area under the receiver operating characteristics–ROC–curve (AUC), and estimates of sensitivity and specificity when data were dichotomized at the usual positivity threshold. For these analyses, TB infection was defined as children with LTBI and TB contact or active TB. The non TB infection group was defined as healthy contacts or children with LTBI and no TB contact.

Results of QF-TB-IT were compared in active TB children according to culture, family contact and chest X-ray using the signed Wilcoxon rank sum test, while the proportions of QF-TB-IT and positive cultures were compared in these patients by the Liddell's exact test for paired data.

The association between TST induration diameter and QF-TB-IT was assessed using Spearman rank correlation coefficient and the agreement between both dichotomized tests by the kappa statistics.

To analyze the evolution of QF-TB-IT during treatment, values were compared between day 0 and day 10 and, then, between day 0 and the date of the end of chemoprophylaxis, using paired Wilcoxon tests (quantitative values). To avoid an increase of the type I error rate, dichotomized test values were compared between these time points using Liddell's test only if a significant difference was obtained on the quantitative values.

All tests were two-sided, and p-values <0.05 were regarded as indicating statistical significance. Analyses were carried out using R 2.6.2 statistical software.

## Results

### 1. Patients

A total of 131 children were included in 3 Parisian Pediatric Hospitals and 2 hospitals with pediatric wards located in the North suburb of Paris. The study participants lived in the catchment areas served by the different hospitals and where the incidence of TB is between 15 and ∼100 per 100,000 inhabitants [28], [29].

Two children were eventually excluded from the complete analysis due to insufficient clinical data and follow-up leaving 129 children: 98 patients (healthy contacts, LTBI and active TB, see below) and 31 controls (Table 1, Data S1). The median age was 7.4 years [range 2 months to 17 y]. Age distribution was not significantly different in the four groups (p = 0.073) (Table 1). Half were male and half were females. 69% were born in France. The vast majority of the children were BCG vaccinated (91%). 69% of the children had a known TB contact, which was in 40% of cases a family member (Table 1). This is in comparison with 45% of known family members with a previous history of tuberculosis (Table 1). One control child had a known family contact (a cousin with a TST conversion only) but was finally categorized by the treating clinician as a control because of a negative TST value (0 mm), an absence of clinical symptoms and a normal chest X-Ray. 83% of the children had TST induration measured. However, when considering only the 98 patients for which TST induration diameter was one of the inclusion criteria, 97% (95/98) had a TST value, of whom 81.3% were positive (TST≥10 mm) (Data S1). In addition a known TST conversion was observed in 54% of the children (Table 1).

**Table 1:** Characteristics of the enrolled children

Variable	Controls	Healthy contacts	Latent TB	Active TB	Total
No. patients	31	12	54	32	129
Gender, no. (%)					
F	14 (45)	6 (50)	26 (48)	17 (53)	63 (49)
M	17 (55)	6 (50)	28 (52)	15 (47)	66 (51)
Median age (range), ys	6.5 (0.3 to 15.6)	2.4 (0.2 to 16.9)	9.2 (0.5 to 17)	7.3 (0.9 to 17.1)	7.4 (0.2 to 17)
Country of birth, no. (%)					
France	25 (81)	9 (75)	37 (69)	18 (56)	89 (69)
Southern Europe and Mediterranean	0 (0)	3 (25)	2 (4)	1 (3)	6 (5)
Africa	4 (13)	0 (0)	9 (17)	8 (25)	21 (16)
East Asia	0 (0)	0 (0)	3 (6)	3 (9)	6 (5)
Caribbean islands	0 (0)	0 (0)	1 (2)	1 (3)	2 (2)
Unknown	2 (6)	0 (0)	2 (4)	1 (3)	5 (4)
BCG vaccination, no. (%)					
Yes	29 (94)	10 (83)	50 (93)	29 (91)	118 (91)
No	2 (6)	2 (17)	1 (2)	0 (0)	5 (4)
Unknown	0 (0)	0 (0)	3 (6)	3 (9)	6 (5)
Health coverage, no. (%)	28 (100)	11 (100)	48 (96)	25 (96)	112 (97)
Family contact, no. (%)					
Yes	1 (3)	7 (58)	32 (59)	12 (38)	52 (40)
No	11 (35)	1 (8)	11 (20)	14 (44)	37 (29)
Unknown	19 (61)	4 (33)	11 (20)	6 (19)	40 (31)
TST induration diameter (mm), no. (%)					
No. (%) tested	12 (39)	11 (92)	53 (98)	31 (97)	107 (83)
0–4	6 (50)	6 (55)	2 (4)	4 (13)	18 (17)
5–9	1 (8)	0 (0)	1 (2)	0 (0)	2 (2)
10–15	4 (33)	3 (27)	16 (30)	9 (29)	32 (30)
>15	1 (8)	2 (18)	34 (64)	18 (58)	55 (51)
TST conversion, no. (%)					
No	22 (71)	8 (67)	9 (17)	9 (28)	48 (37)
Yes	1 (3)	4 (33)	44 (81)	21 (66)	70 (54)
Unknown	8 (26)	0 (0)	1 (2)	2 (6)	11 (9)
Previous tuberculosis					
Child	0 (0)	0 (0)	1 (2)	1 (3)	2 (2)
Family	3 (10)	6 (50)	36 (67)	13 (41)	58 (45)
Unknown	6 (19)	1 (8)	5 (9)	5 (16)	17 (13)

### 2. Clinical, radiological and bacteriological results

The study population was separated into 4 groups: controls (31 children), healthy contacts (HC) (12 children), LTBI (54 children) and active TB (32 children). This classification was defined by national guidelines and inclusion criteria described above. Chest radiographic abnormalities were observed in 78%, 3.7% and 8.3% of children with active TB, LTBI and HC respectively. 1 of 12 HC (8.3%) had an abnormal chest X-ray (pneumomediastinum), and 2 out of 54 (3.7%) LTBI showed evidence of healed non tuberculous infection. All the latently infected children, and the HC, were acid fast smear and culture negative on all specimens tested (Table 2). By comparison, 48% of children with active TB had culture positive specimens for *M. tuberculosis* of whom 19% were acid fast bacilli (AFB) smear positive (Table 2). All isolates were fully sensitive to all drugs prescribed. 64% of children with active TB had pulmonary tuberculosis, 36% had extrapulmonary tuberculosis. AFB smears were positive in more children with pulmonary TB (32%) than in children with extrapulmonary TB (8%). Similarly, a higher proportion of children with pulmonary TB were culture positive (58%) compared to extrapulmonary disease (33%). All children with active TB were treated for a median duration time of 6 months (Table 2). One patient had to stop treatment after 2 months due to acute drug related hepatitis. All of the LTBI children received chemoprophylaxis for a median duration time of 3 months (Table 2). 83% HC also received chemoprophylaxis for a median duration time of 3 months (Table 2).

**Table 2:** Clinical, microbiological and treatment characteristics of the children population

Variable	Healthy contacts	Latent TB	Active TB	Total
No. patients	12	54	32	98
Chest X-ray, no. (%)				
Negative	10 (83)	50 (93)	7 (22)	67 (68)
Positive	1 (8)	2 (4)	25 (78)	28 (29)
Not done or unknown	1 (8)	2 (4)	0 (0)	3 (3)
Bacteriology, no. (%)				
Not done	2 (17)	17 (31)	1 (3)	20 (20)
Smear				
Negative	10 (100)	37 (100)	25 (81)	72 (92)
Positive	0 (0)	0 (0)	6 (19)	6 (8)
Culture				
Negative	10 (100)	36 (100)	16 (52)	62 (81)
Positive	0 (0)	0 (0)	15 (48)	15 (19)
Treatment, no. (%)	10 (83)	54 (100)	32 (100)	96 (98)
Median treatment duration (range), months	3 (1 to 3)	3 (2 to 6)	6 (2 to 12)	3 (1 to 12)
Treatment outcome, no. (%)				
Cure or treatment completed	7 (70)	46 (85)	26 (81)	79 (82)
Treatment stop	0 (0)	0 (0)	1 (3)	1 (1)
Loss to follow-up	3 (30)	8 (15)	2 (6)	13 (14)
Transfer or still under treatment	0 (0)	0 (0)	3 (9)	3 (3)

### 3. QF-TB-IT IFNγ values at enrolment

**Figure 1:**
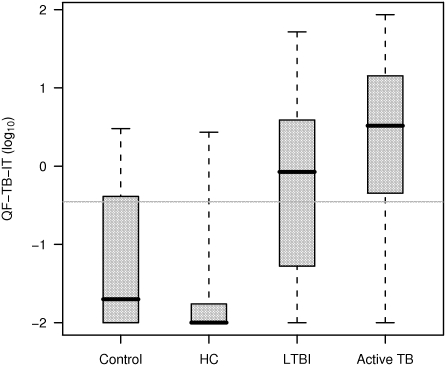
Distribution of QF-TB-IT (in log_10_) for all groups of patients. QF-TB-IT values are displayed as a log scale, and null values were arbitrarily set at 0.01 (below the smallest observed non-null value). Box and whiskers plots present the median, first and third quartile of the distribution (box) and outer whiskers extend the whole range of data. QF-TB-IT positivity was defined as values equal or superior to log_10_ (0.35) which correspond to -0.46 (grey line).

In our study, TST was used to support the inclusion of children into any of the diagnostic categories. By so, we presented data regarding the behavior of QF-TB-IT only, for each category. 30%, 9%, 58% and 78% of controls, HC, LTB infected and active TB children respectively were QF-TB-IT positive (Data S1). The four groups differed significantly in QF-TB-IT IFNγ values (Table 3) (p<0.0001, Kruskal-Wallis test). Pairwise group comparisons showed that QF-TB-IT IFNγ distributions were significantly different between all groups, except between controls and HC, with in particular a significant difference in IFNγ values between LTBI and active TB (p = 0.010, Wilcoxon rank sum test) (figure 1). These results remained unchanged when adjusting analyses for age using analysis of covariance on log-transformed data.

**Table 3:** QF-TB-IT distribution among all groups of patients.

		QF-TB-IT (IU/ml)		
	No* patients	No* missing data	Median (Q_1_–Q_3_)	No (%)≥0.35
Controls	31	1	0.02 (0–0.40)	9 (30%)
Healthy contact	12	1	0 (0–0.02)	1 (9%)
Latent TB	54	2	0.85 (0.06–3.75)	30 (58%)
Active TB	32	0	3.28 (0.51–13.97)	25 (78%)

(*): number

**Figure 2:**
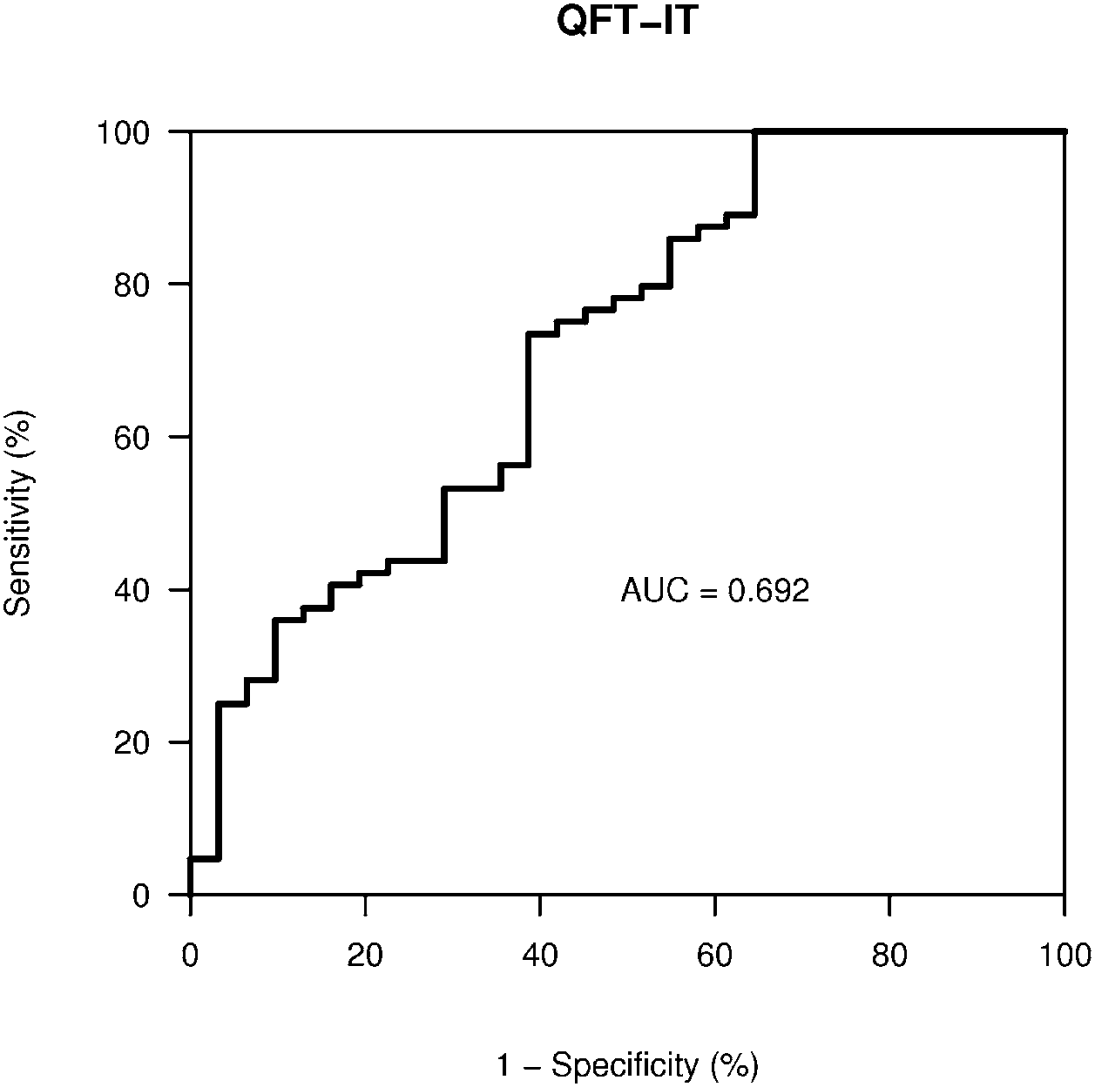
ROC curve of QF-TB-IT to predict TB infection in LTBI with known family contact and active TB children.

We evaluated the performance of QF-TB-IT as a marker of TB infection, defined as LTBI children with a known family TB contact (32 children) and active TB children (32 children) versus healthy contacts (12 children) or children with LTBI and no TB contact (22 children). The area under the ROC curve was 0.692 (figure 2). When using the supplier defined positivity threshold at 0.35, this resulted in a sensitivity of 69% (95%CI 56% to 80%) and a specificity of 61% (42% to 78%).

**Figure 3:**
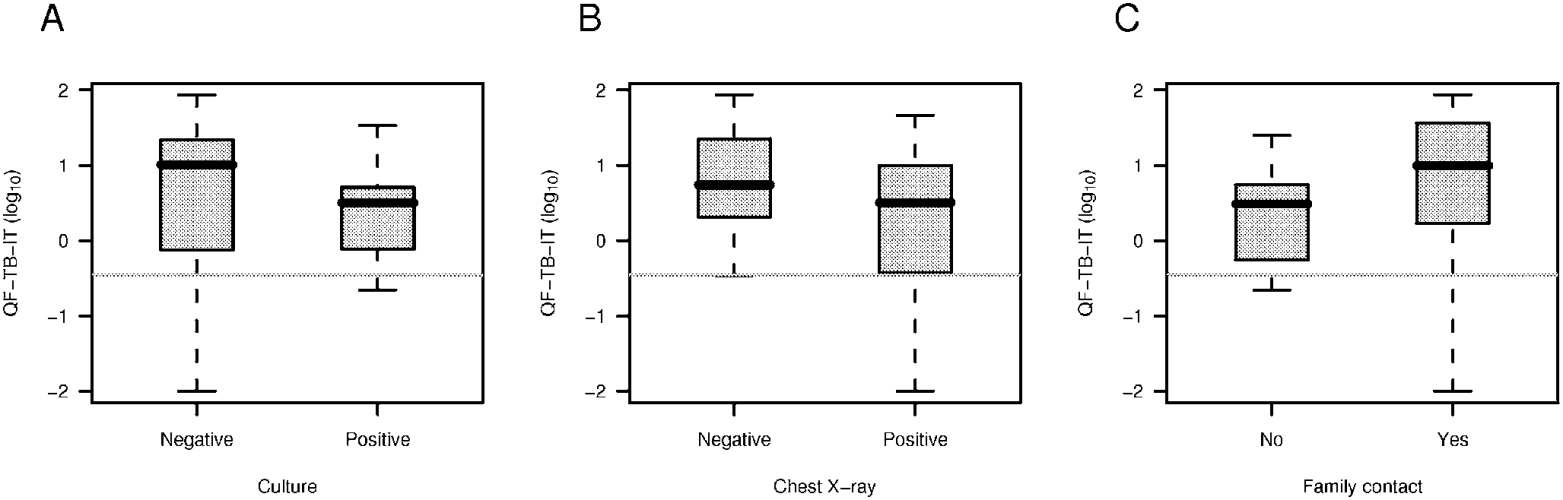
Distribution of QF-TB-IT (in log_10_) for culture positive and culture negative active TB children (A), for positive and negative chest X-Ray (B) and for known TB family contact (C). Box and whiskers plots present the median, first and third quartile of the distribution (box) and outer whiskers extend the whole range of data. QF-TB-IT values were displayed on a log scale, and null values were arbitrarily set at 0.01 (below the smallest observed non-null value).

Proportion of positive culture in active TB children was 48% (15/31). This rate was significantly lower than the rate of positive QF-TB-IT in these children (p = 0.021, Liddell test). Finally, QF-TB-IT values were not significantly different in active TB children with positive and negative culture (p = 0.26, figure 3A), positive and negative chest X-ray (p = 0.31, figure 3B) or presence of family contact (p = 0.18, figure 3C).

### 4. TST, QF-TB-IT correlation, age distribution

**Figure 4:**
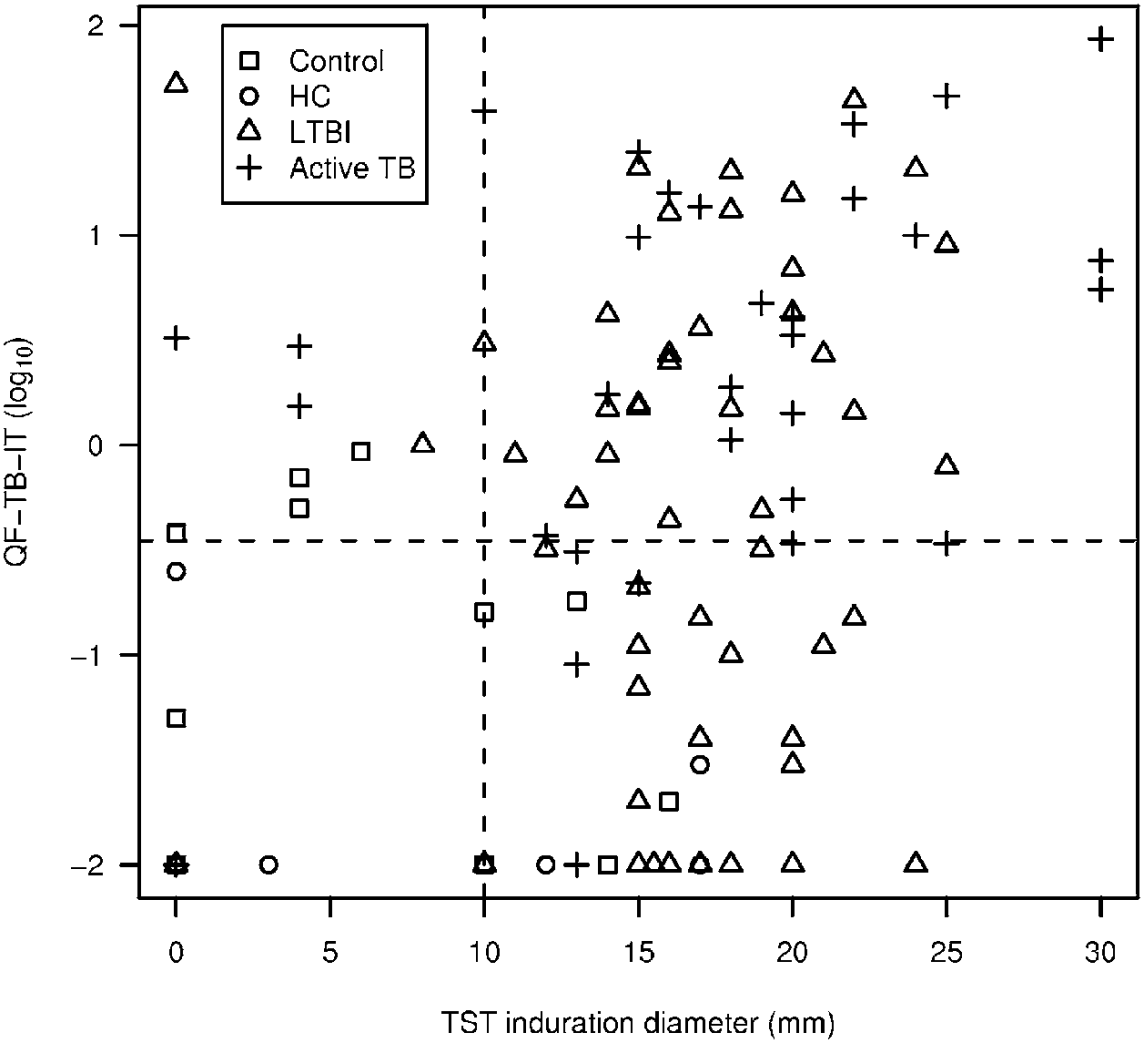
Scatter-plot of TST induration diameter (in mm) and QF-TB-IT (in log_10_) in all patients. QF-TB-IT values were display on a log scale, and null values were arbitrarily set at 0.01 (below the smallest observed non-null value). TST positivity was defined as values equal or superior to 10 mm (grey line) QF-TB-IT positivity was defined as values equal or superior to log_10_ (0.35) which correspond to -0.46 (grey line).

TST induration diameter and QFT-IT were significantly correlated (Spearman rank correlation coefficient 0.39, p<0.0001) (figure 4). However, when using the conventional thresholds (10 mm and log_10_ (0.35) IU/ml), the tests poorly agreed (kappa statistics 0.08). TST induration was also found to be correlated with age (Spearman rho 0.34, p = 0.0003), but this was not the case for QF-TB-IT (Spearman rho 0.04, p = 0.66) (data not shown).

### 5. Kinetics of the IFN-γ response throughout treatment for HC, LTBI and active TB children

**Figure 5:**
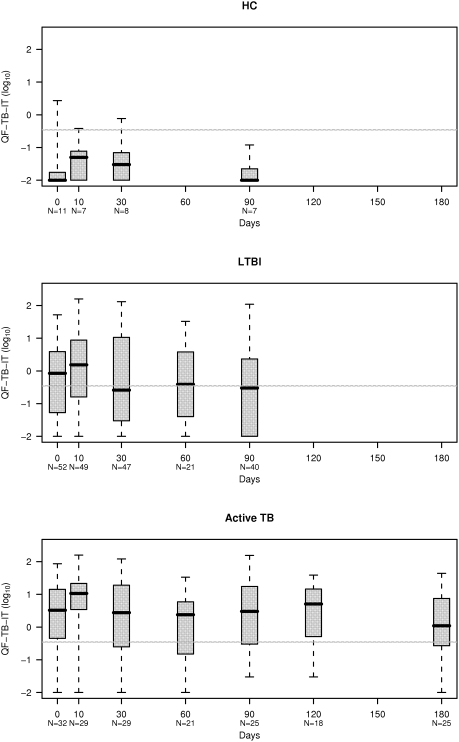
Distribution of QF-TB-IT (in log_10_) during follow-up, in Healthy Contacts (HC), Latent TB Infection (LTBI) and Active TB Children. QF-TB-IT values are displayed on a log scale, and null values were arbitrarily set at 0.01 (below the smallest observed non-null value). QF-TB-IT positivity was defined as values equal or superior to log_10_ (0.35) which correspond to −0.46 (grey line). LTBI and active TB children presented a significant variation between day 0 and day 10 under treatment (p = 0.035, paired Wilcoxon rank test). In LTBI, QF-TB-IT values were significantly lower at day 90 as compared to day 0 (p = 0.042, paired Wilcoxon rank test). In active TB, QF-TB-IT values were significantly lower at day 180 as compared to day 0 (p = 0.041, paired Wilcoxon rank test).

The distribution of QF-TB-IT values during follow-up (Data S2) is shown in figure 5 for each category. LTBI and active TB children produced a significant variation between day 0 and day 10 of treatment (p = 0.035, paired Wilcoxon rank test). This is exemplified by significant higher IFN-γ values after 10 days of treatment (figure 5, see also figure S1). This variation was similar for LTBI and active TB children (p = 0.95, Wilcoxon rank-sum test).

When considering all enrolled children (except controls and HC) who underwent QF-TB-IT testing at day 0, 10 and 30 (78 out of 86); 29 were negative at day 0. 14 out of 29 became positive at day 10. Five were still positive at day 30 and 9 became negative at day 30. 15 out of 29 were negative at day 0 and day 10. Two out of 15 became positive at day 30. If we now considered all cases with at least one positive value at day 0, day 10 or day 30, 44 LTBI children out of 54 had at least one positive sample and 29 active TB children out of 32 had at least one positive sample.

Thirty eight LTBI children had QF-TB-IT at day 0 and day 90. QF-TB-IT IFNγ values were significantly lower at day 90 as compared to day 0 (p = 0.042, paired Wilcoxon rank test) (figure 5), but the numbers of patients with positive QF-TB-IT values (≥0.35 IU/ml) at day 0 (25 out of 38, 66%) and at day 90 (19 out of 38, 50%) were not significantly different (p = 0.21, Liddell test).

Twenty five active TB children had QF-TB-IT values at day 0 and day 180. QF-TB-IT IFNγ values were significantly lower at day 180 as compared to day 0 (p = 0.041, paired Wilcoxon rank test) (figure 5), however, as for LTBI patients, the numbers of patients with positive QF-TB-IT values (≥0.35 IU/ml) at day 0 and day 180 were not significantly different (21 out of 25 [84%] vs18 out of 25 [72%], p = 0.51, Liddell test).

## Discussion

A key question regarding IGRA and one often asked by clinicians is whether IGRA might replace the TST. Longitudinal studies have correlated the size of induration with future risk of active TB [30]–[32]. However, TST interpretation on a background of in excess of 90% of individuals with a BCG vaccine has triggered the need to develop a model for efficient screening to avoid an excessive number of false-positive subjects and subsequent overtreatment during contact tracing investigations [32]–[34 and our study]. This problem is further emphasized when screening children, as they represent the most susceptible of the infected contacts for future development of active TB. This is shown in our prospective study where 46% of HC were TST positive as classified by clinicians, and 83% received chemoprophylaxis. By comparison only 9% of HC had a positive QF-TB-IT result.

Enrolment of our children started in October 2005, on a background of in excess of 90% of individuals with a BCG vaccine and at a time when IGRA were not yet licensed in France. However, discussion and proposals were underway to move towards a new TB control strategy with the abolition of BCG vaccination, and it was important to evaluate the impact of IGRA (in our study QF-TB-IT) on TB diagnosis in children. Up until this time, studies examining IGRA in children in both high [12], [16], [17], [19], [20], [22], [23] or low burden [11], [14], [15], [18], [21] regions of tuberculosis infection have demonstrated a higher accuracy of IGRA for TB except for the Connell *et al* and Nicol *et al*. studies [14], [23].

Significantly higher IFNγ values were obtained from children with active TB as compared to LTBI children. However, there was an overlap in QF-TB-IT IFNγ values between these categories (see figure 1), not allowing a clear separation between both clinical presentations. But this represents a major issue for IGRA [33], [36]. In comparison, an adult pilot study using an ESAT-6 ELISPOT approach, showed that lymph node TB and self healed culture negative TB patients had higher frequencies of ESAT-6 specific CD4 T-cells than culture positive TB patients [37]. This might indicate that replicating *M. tuberculosis* and its antigen burden finely control the level of T cell-IFN-γ responses [38]–[40]. One might expect a lower burden of *M. tuberculosis* antigens in our LTBI children or in culture negative active TB children, and by correlation a higher IFN-γ response, as seen recently [37], [41]. Despite the fact that several culture negative active TB children had very high QF-TB-IT values (not shown), the geometric mean was no different when we compared it to the geometric mean calculated from culture positive active TB children IFN-γ values, although a trend was observed (see figure 3A).

Another hypothesis might be that above a certain level of replicating mycobacteria, T-cell responses are not detectable *in vitro* as T-cells might be exhausted or sequestered at the site of infection. This argument was often used to explain the absence of a TST response in patients with advanced tuberculosis. It represents one of the difficulties encountered by IGRA, with negative results observed in culture confirmed tuberculosis [10], [12], [13], [23 and our study]. The dynamics of the T-cell response during active or latent TB was difficult to analyse and thereby to understand when only the TST was available, as repeated intradermal injections of tuberculin can caused a boosting effect [31], artificially enhancing the TST response. By comparison, IGRA, based on a simple venous puncture, might be repeated without interfering with the existing *in vivo* T-cell activity. Setting up a prospective study to investigate the effect of preventive and curative therapy on the IGRA response over time has allowed us to detect an increase in the IFN-γ response as soon as 10 days after institution of therapy, whether it was for LTBI or active TB. This is the first time to our knowledge that the IFN-γ response has been analyzed so shortly after institution of treatment, and that such a rebound in the IFN-γ response is described in children. This has not been observed before in children mainly because the delay between the first and the second performed IGRA was too long; more than 1, 3, 6, 12 and 18 months depending on the protocol [13], [42]. However, an increase in the PPD, but not ESAT-6 or CFP-10 ELISPOT response was observed in children after 1 month treatment in the study by Nicol *et al*. [13]. By comparison, Wilkinson *et al*. demonstrated a 1.8 fold increased in the ESAT-6/CFP-10 ELISPOT response at 26 days in LTBI and treated adults [43]. This was not observed in another study but the first sampling after starting treatment was 5 weeks [37]. This rebound is clearly significant in our study when comparing QF-TB-IT values at day 0 and at day 10.

Although this result might be expected for active disease, it is more surprising for LTBI. This rebound during chemoprophylaxis observed in LTBI children might confirm the potential of the IGRA to detect recently acquired infection and the presence of low level of replicative bacteria. It could also demonstrate all the difficulties of classifying children either as latently infected or as presenting with active disease. Fourteen LTBI and active TB children with a negative QF-TB-IT response at day 0 rapidly developed a positive response after 10 days of preventive or curative therapy. In addition, two more produced a positive response after one month of preventive therapy. Strikingly, active TB children with a negative QF-TB-IT response at day 0, but a positive a day 10, were all culture positive. For each child showing an increase in IFNγ concentration at day 10 and day 30 as compared to day 0, the second test result was higher than 16% of the first QF-TB-IT IFNγ result, which is the threshold limit potentially defined as true conversion [44]. As all children were enrolled and treated similarly, it is unlikely that previous intradermal injection of tuberculin, often performed several weeks before, would have been responsible for this increase [45]. In fact (see below) several children classified as LTBI or with active TB had negative IFNγ values throughout the follow-up study despite being referred because of a TST switch or a TST induration diameter above 10 mm. Increases in IFNγ values due to previous intradermal injection of tuberculin are often low [45], as compared to the increase observed in our study due to treatment (see figure 5, active TB children).

The results we present here either satisfy the hypothesis of T-cell sequestration or exhaustion and that killing bacteria lowers the antigenic burden and restores active T-cells detectable *in vitro*, as seen in TB paradoxical reactions [46], [47]; or indicate that killing bacteria release cytosolic stores full of ESAT-6 and CFP-10 antigens into the macrophage resulting in a transient increase in antigen presentation stimulating a larger T-cell response [42], [48]. In addition, a significant decline in IFNγ response was observed in LTBI and active TB treated patients, when comparing quantitative IFNγ level measured by QF-TB-IT at day 0 and 90 or 180 for LTBI and active TB children respectively. Similar declines were also observed at 3 and 6 months in two other monitoring studies [13], [42], confirming the decrease of the antigenic and bacterial load due to preventive or curative therapy.

Such an early rebound might allow confirmation of the diagnosis, whether it is LTBI or active TB. It needs a prospective study with a treatment decision using IGRA values obtained between day 0 and day 30. In addition, such a decline in response may help clinicians to monitor the effect of preventive and curative therapy on latent or active infection [49], [50].

Finally it demonstrates that the use of IGRA as a one-shot strategy similar to the TST approach might not be the correct strategy, as several LTBI or active TB children will be missed [23, our study]. Similar results were recently obtained in a high incidence area for TB [23]. T-SPOT-TB, when compared to TST for the detection of active tuberculosis in children, demonstrated a poorer sensitivity as compared to TST for a combined group of culture-confirmed and clinically probable tuberculosis, indicating that T-SPOT-TB can not be used to exclude active disease [23]. It emphasizes the need for a follow-up strategy as shown in our study, with an increase in QF-TB-IT sensitivity when combining day 0, 10 and 30 results.

In conclusion, our results have shown the importance of carefully recording the dynamics of the IFNγ response in children. We need to follow up on this study by implementing IGRA in any children case contact study or in high risk children population. In addition, repeating IGRA whether the first value is positive or negative will provide reassurance to the clinician in his treatment choice. This reinforces the hypothesis that high and/or rising levels of IFNγ in response to ESAT-6/CFP-10/Tb7.7 antigens might serve as a prognostic marker, thus allowing the targeting of those children in this category for treatment.

## Supporting Information

Data S1 Flowchart of study children at enrollment (day 0)
(0.04 MB DOC)

Data S2 Flowchart of the kinetic study
(0.04 MB DOC)

Figure S1 Children individual kinetics throughout the follow-up study
(0.08 MB TIF)

## References

[1] Global tuberculosis control: surveillance, planning, financing..

[2] Shingadia D, Novell V (2003). Diagnosis and treatment of tuberculosis in children.. The Lancet Infect Dis.

[3] Marais BJ, Pai M (2007). Recent advances in the diagnosis of childhood tuberculosis.. Arch Dis Child.

[4] Guidance for national tuberculosis programmes on the management of tuberculosis in children..

[5] Murray CJ, Styblo K, Rouillon A (1990). Tuberculosis in developing countries: burden, intervention and cost.. Bull Int Union Tuberc Lung Dis.

[6] van Rie A, Beyers N, Gie RP, Kunneke M, Zietsman L (1999). Childhood tuberculosis in an urban population in South Africa: burden and risk factor.. Arch Dis Child.

[7] Simonney N, Bourrillon A, Lagrange PH (2000). Analysis of circulating immune complexes (CICs) in childhood tuberculosis: levels of specific antibodies to glycolipid antigens and relationship with serum antibodies.. Int J Tuberc Lung Dis.

[8] Lagrange PH, Simonney N, Wargnier A, Herrmann JL (2001). Usefulness of serological tests in childhood TB.. Pediatr Pulmonol.

[9] Antoine D, Che D (2008). Cases of tuberculosis diseases notified in France in 2006.. Bull Epid Hebdomadaire.

[10] Pai M, Riley LW, Colford JM (2004). Interferon-gamma assays in the immunodiagnosis of tuberculosis: a systematic review.. Lancet Infect Dis.

[11] Ewer K, Deeks J, Alvarez L, Bryant G, Waller S (2003). Comparison of T-cell-based assay with tuberculin skin test for diagnosis of Mycobacterium tuberculosis infection in a school tuberculosis outbreak.. Lancet.

[12] Lieberschuetz S, Bamber S, Ewer K, Deeks J, Pathan AA (2004). Diagnosis of tuberculosis in South African children with a T-cell-based assay: a prospective cohort study.. Lancet.

[13] Nicol MP, Piennar D, Wood K, Eley B, Wilkinson RJ (2005). Enzyme-linked immunospot assay responses to early secretary antigenic target, culture filtrate protein 10, and purified protein derivative among children with tuberculosis: implications for diagnosis and monitoring of therapy.. Clin Infect Dis.

[14] Connell TG, Curtis N, Ranganathan SC, Buttery JP (2006). Performance of a whole blood interferon gamma assay for detecting latent infection with *Mycobacterium tuberculosis* in children.. Thorax.

[15] Connell TG, Ritz N, Paxton GA, Buttery JP, Curtis N (2008). A three-way comparison of tuberculin skin testing, QuantiFERON-TB Gold and T-SPOT.TB in children.. PLOS One.

[16] Tsiouris SJ, Austin J, Toro P, Coetzee D, Weyer K (2006). Results of a tuberculosis-specific IFN-γ assay in children at high risk for tuberculosis infection.. Int J Tuberc Lung Dis.

[17] Nakaoka H, Lawson L, Squire SB, Coulter B, Ravn P (2006). Risk for tuberculosis among children.. Emerg Infect Dis.

[18] Detjen AK, Keil T, Roll S, Hauer B, Mauch H (2007). Comparison of QFT Gold In-Tube and T-Spot in Children: Interferon-gamma release assays improve the diagnosis of tuberculosis and non tuberculous mycobacterial disease in children in a country with a low incidence of tuberculosis.. Clin Infect Dis.

[19] Okada K, Mao TE, Mori T, Miura T, Sugiyama T (2007). Performance of an interferon-gamma release assay for diagnosing latent tuberculosis infection in children.. Epidemiol Infect.

[20] Dogra S, Narang P, Mendiratta DK, Chaturvedi P, Reingold AL (2007). Comparison of a whole blood interferon-γ assay with tuberculin skin testing for the detection of tuberculosis infection in hospitalized children in rural India.. J Infect.

[21] Higuchi K, Harada N, Mori T, Sekiya Y (2007). Use of QuantiFeron® -TB Gold to investigate tuberculosis contacts in high school.. Respirology.

[22] Hill PC, Brookes RH, Adetifa IM, Fox A, Jackson-Sillah D (2006). Comparison of enzyme-linked immunospot assay and tuberculin skin test in healthy children exposed to *Mycobacterium tuberculosis*.. Pediatrics.

[23] Nicol MP, Davies M-A, Wood K, Hatherill M, Workman L (2008). A comparison of T-SPOT.*TB* and tuberculin skin test for the evaluation of young children at high risk for tuberculosis in a community setting. Pediatrics.. Accepted for publication.

[24] Lalvani A, Millington KA (2007). T cell-based diagnosis of childhood tuberculosis infection.. Curr Op Infect Dis.

[25] Herrmann JL, Simonney N, Bergeron A, Ducreux-Adolphe N, Porcher R (2008). IFN gamma and antibody responses among French nurses during a tuberculosis contact tracing investigation.. Pathol Biol (Paris) [Apr 3, Epub ahead of print].

[26] Delacourt C, Albertini M, Decludt B, Scheinmann P, Marguet C (2004). What examinations are necessary in an exposed, asymptomatic child with a positive tuberculin skin test and normal chest x-ray ?. [in French] Rev Mal Respir.

[27] Conseil Superieur d'Hygiene Publique de France. TB case investigation: practice recommendations [in French]. [Created 2006 Mar; accessed 2008 Mar 14].. http://www.sante.gouv.fr/htm/dossiers/tuberculose/reco_cshpf.pdf.

[28] Antoun F, Mallet H-P (2005). La tuberculose à Paris en 2003, situation actuelle et contribution du Service de lutte anti-tuberculose.. Bull Epid Hebdomadaire.

[29] Antoine D, Che D (2007). Cases of tuberculosis diseases notified in France in 2005.. Bull Epid Hebdomadaire.

[30] Grzybowski S, Barnett GD, Styblo K (1975). Contacts of cases of active pulmonary tuberculosis.. Bull Int Union Tuberc.

[31] Watkins RE, Brennan R, Plant AJ (2000). Tuberculin reactivity and the risk of tuberculosis: a review.. Int J Tuberc Lung Dis.

[32] Stout JE, Menzies D (2008). Predicting tuberculosis: does IGRA tell the tall?. Am J Respir Crit Care Med.

[33] Diel R, Loddenkemper R, Meywald-Walter K, Niemann S, Nienhaus A (2008). Predictive value of a whole blood IFN-gamma assay for the development of active tuberculosis disease after recent infection with *Mycobacterium tuberculosis*.. Am J Respir Crit Care Med.

[34] Aissa K, Madhi F, Ronsin N, Delarocque F, Lecuyer A (2008). Evaluation of a model for efficient screening of tuberculosis contact subjects.. Am J Respir Crit Care Med.

[35] Andersen P, Doherty TM, Pai M, Weldingh K (2007). The prognosis of latent tuberculosis: can disease be predicted?. Trends Mol Med.

[36] Doherty TM, Demissie A, Olobo J, Wolday D, Britton S (2002). Immune responses to the *Mycobacterium tuberculosis*-specific antigen ESAT-6 signal subclinical infection among contacts of tuberculosis patients.. J Clin Microbiol.

[37] Pathan AA, Wilkinson KA, Klenerman P, McShane H, Davidson RN (2001). Direct *ex vivo* analysis of antigen-specific IFN-gamma-secreting CD4 T-cells in *Mycobacterium tuberculosis*-infected individuals: associations with clinical disease state and effect of treatment.. J Immunol.

[38] Vordermeier HM, Chambers MA, Cockle PJ, Whelan AO, Simmons J (2002). Correlation of ESAT-6-specific gamma interferon production with pathology in cattle following *Mycobacterium bovis* BCG vaccination against experimental bovine tuberculosis.. Infect Immun.

[39] Buddle BM, Wedlock DN, Denis M, Skinner MA (2005). Identification of immune response correlates for protection against bovine tuberculosis.. Vet Immunol Immunopathol.

[40] Langermans JA, Doherty TM, Vervenne RA, van der Laan T, Lyashchenko K (2005). Protection of macaques against *Mycobacterium tuberculosis* infection by a subunit vaccine based on a fusion protein of antigen 85B and ESAT-6.. Vaccine.

[41] Vekemans J, Lienhardt C, Sillah JS, Wheeler JG, Lahai GP (2001). Tuberculosis contacts but not patients have higher gamma interferon responses to ESAT-6 than do community controls in The Gambia.. Infect Immun.

[42] Ewer K, Millington KA, Deeks JJ, Alvarez L, Bryant G (2006). Dynamic antigen-specific T-cell responses after point-source exposure to *Mycobacterium tuberculosis*.. Am J Respir Crit Care Med.

[43] Wilkinson KA, Kon OM, Newton SM, Meintjes G, Davidson RN (2006). Effect of treatment of latent tuberculosis infection on the T cell response to Mycobacterium tuberculosis antigens.. J Infect Dis.

[44] Veerapathran A, Joshi R, Goswami K, Dogra S, Moodie EE (2008). T-cell assays for tuberculosis infection: deriving cut-offs for conversions using reproducibility data.. PLoS ONE.

[45] Leyten EM, Prins C, Bossink AW, Thijsen S, Ottenhoff TH (2007). Effect of tuberculin skin testing on *Mycobacterium tuberculosis*-specific interferon-gamma assay.. Eur Resp J.

[46] Wilkinson RJ, Vordermeier HM, Wilkinson KA, Sjölund A, Moreno C (1998). Peptide-specific T cell response to *Mycobacterium tuberculosis*: clinical spectrum, compartmentalization, and effect of chemotherapy.. J Infect Dis.

[47] Bourgarit A, Carcelain G, Martinez V, Lascoux C, Delcey V (2006). Explosion of tuberculin-specific Th1-responses induces immune restoration syndrome in tuberculosis and HIV co-infected patients.. AIDS.

[48] Pym AS, Brodin P, Majlessi L, Brosch R, Demangel C (2003). Recombinant BCG exporting ESAT-6 confers enhanced protection against tuberculosis.. Nat Med.

[49] Lalvani A (2004). Counting antigen-specific T cells: a new approach for monitoring response to tuberculosis treatment?. Clin Infect Dis.

[50] Carrara S, Vincenti D, Petrosillo N, Amicosante M, Girardi E (2004). Use of a T cell-based assay for monitoring efficacy of antituberculosis therapy.. Clin Infect Dis.

