# Supplementary material for: Temporal Dynamics of Interferon Gamma Responses in Children Evaluated for Tuberculosis
Source: PLoS One. 2009 Jan 6;4(1):e4130. doi: 10.1371/journal.pone.0004130 (PMC2607538; doi:10.1371/journal.pone.0004130)
Supplement: Data S1 — Flowchart of study children at enrollment (day 0) (0.04 MB DOC) [file pone.0004130.s001.doc]

Children with activeTB

n=32

Children with LTBI

n=54

Healthy contact Children

n=12

Controls

n=31

TST done

n = 31

TST not

done n = 1

QF-TB done

n = 32

No sample

received n = 0

TST done

n = 53

TST not

done n = 1

QF-TB done

n = 52

No sample

received n = 2

TST done

n = 11

TST not

done n = 1

QF-TB done

n = 11

No sample

received n = 1

QF-TB done

n = 30

No reading

n = 0

Positive

n = 27 (87)*

Negative

n = 4

Positive

n = 50 (94)*

Negative

n = 3

Positive

n = 5 (45)*

Negative

n = 6

Positive

n = 25 (78)*

Negative

n = 7

Positive

n = 30 (58)*

Negative

n = 22

Positive

n = 1 (9)*

Negative

n = 10

Positive

n = 9 (30)*

Negative

n = 21

No reading

n = 0

No reading

n = 0

(*) percentage of positivity

**Data S1: Flow chart of study children at enrollment (day 0)**
