# Supplementary material for: Temporal Dynamics of Interferon Gamma Responses in Children Evaluated for Tuberculosis
Source: PLoS One. 2009 Jan 6;4(1):e4130. doi: 10.1371/journal.pone.0004130 (PMC2607538; doi:10.1371/journal.pone.0004130)
Supplement: Data S2 — Flowchart of the kinetic study (0.04 MB DOC) [file pone.0004130.s002.doc]

Children with activeTB

N = 32

Day 0

n = 32

Day 10

n = 29

Day 30

n = 29

Day 60

n = 21

Day 90

n = 25

Day 180

n = 25

Positive

n = 25 (78)*

Positive

n = 25 (86)

Positive

n = 20 (69)

Positive

n = 14 (67)

Positive

n = 18 (72)

Positive

n = 18 (72)

Children with LTBI

N = 54

Day 0

n = 52

Day 10

n = 49

Day 30

n = 47

Day 60

n = 21

Day 90

n = 40

Positive

n = 29 (58)*

Positive

n = 33 (67)

Positive

n = 23 (49)

Positive

n = 11 (52)

Positive

n = 20 (50)

Healthy contacts

N = 12

Day 0

n = 11

Day 10

n = 7

Day 30

n = 8

Day 90

n = 7

Positive

n = 1 (9)

Positive

n = 1 (14)

Positive

n = 1 (13)

Positive

n = 0 (0)

N = number of followed up children

n = number of received and tested QF-TB plasma specimens

(*) = percentage of positive plasma samples for IFNg determination

**Data S2: Flowchart of the kinetic study**
